# Accurate Computation of Thermodynamic Activation Parameters in the Chorismate Mutase Reaction from Empirical Valence Bond Simulations

**DOI:** 10.1021/acs.jctc.3c01105

**Published:** 2023-12-19

**Authors:** Ryan Scott Wilkins, Bjarte Aarmo Lund, Geir Villy Isaksen, Johan Åqvist, Bjørn Olav Brandsdal

**Affiliations:** Hylleraas Centre for Quantum Molecular Sciences, Department of Chemistry, University of Tromsø, N9037 Tromsø, Norway

## Abstract

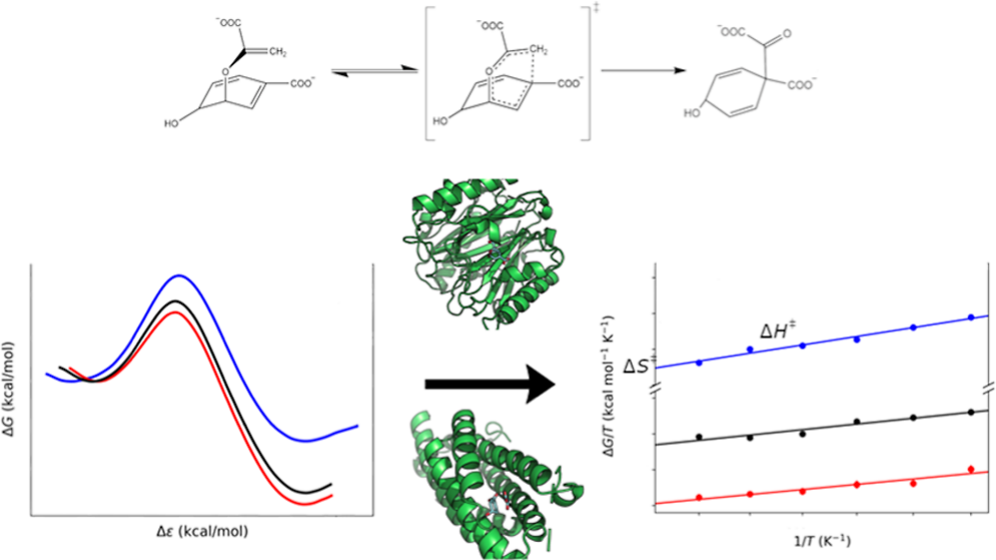

Chorismate mutase (CM) enzymes have long served as model systems for benchmarking new methods and tools in computational chemistry. Despite the enzymes’ prominence in the literature, the extent of the roles that activation enthalpy and entropy play in catalyzing the conversion of chorismate to prephenate is still subject to debate. Knowledge of these parameters is a key piece in fully understanding the mechanism of chorismate mutases. Within this study, we utilize EVB/MD free energy perturbation calculations at a range of temperatures, allowing us to extract activation enthalpies and entropies from an Arrhenius plot of activation free energies of the reaction catalyzed by a monofunctional *Bacillus subtilis* CM and the promiscuous enzyme isochorismate pyruvate lyase of *Pseudomonas aeruginosa*. In comparison to the uncatalyzed reaction, our results show that both enzyme-catalyzed reactions exhibit a substantial reduction in activation enthalpy, while the effect on activation entropy is relatively minor, demonstrating that enzyme-catalyzed CM reactions are enthalpically driven. Furthermore, we observe that the monofunctional CM from *B. subtilis* more efficiently catalyzes this reaction than its promiscuous counterpart. This is supported by a structural analysis of the reaction pathway at the transition state, from which we identified key residues explaining the enthalpically driven nature of the reactions and also the difference in efficiencies between the two enzymes.

## 1 Introduction

A crucial component in understanding the function of enzymes is knowledge of the activation enthalpy–entropy balance and its role in catalyzing the reaction. Current studies of the significance of entropy effects in enzyme catalysis sometimes challenge the celebrated Circe effect hypothesis that ground-state destabilization of the substrate is responsible for the catalytic effect.^1^ Hence, Kazemi et al. proposed that the traditional view of activation entropies being interpreted solely in terms of a loss of translational and rotational entropies upon substrate binding is oversimplified and that a complete representation should also include contributions from the reorganization of the surrounding protein and solvent.^2^ Further understanding of the role of entropy in enzyme catalysis plays an important part in the optimization of catalytic rates and enzyme stability in the rational design of enzymes for industrial and scientific purposes.

**Figure 1:**
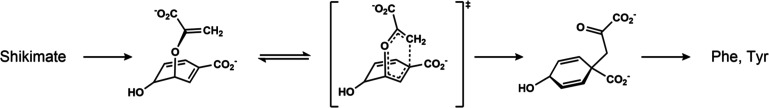
Claisen rearrangement of chorismate to prephenate via a chairlike transition state in CM enzymes.

A common model system used to study the entropy–enthalpy balance in enzymes is chorismate mutase (CM), which has served as a benchmark for QM/MM calculations of enzymatic reactions.^3−6^ CM catalyzes the pericyclic rearrangement of chorismate to prephenate (Figure 1), which is a key step in the shikimate pathway for the biosynthesis of phenylalanine and tyrosine in bacteria, fungi, and higher plants.^7^ There is some debate over the degree to which the entropy plays a role in this reaction. In the AroH-class CM from *Streptomyces aureofaciens*, the enzyme catalyzed reaction exhibits a significantly less negative entropy than the uncatalyzed reaction. This suggests that the conversion of chorismate to prephenate is driven by entropic effects.^8^ This is hypothesized to be due to an electrostatic stabilization of the transition state as the average C_9_–C_1_ distance in the transition state decreases, an energetically costly effect in the water reaction.^9^ While Hur and Bruice considered this to be an example of the formation of a so-called near-attack-complex (NAC), Warshel and co-workers argued that this conformation is the result of transition state stabilization rather than the reason for the catalytic effect of the enzyme.^3^ On the other hand, in the CM reaction catalyzed by the *Bacillus subtilis* CM (BsCM) and the promiscuous enzyme isochorismate pyruvate lyase from *Pseudomonas aeruginosa* (PchB), the activation entropy from experimental studies is comparable to that of the reaction in water, suggesting that entropy plays only a small role in the reaction.^10^ In the latter enzyme, the conversion of chorismate to prephenate is secondary to the conversion of isochorismate to pyruvate and salicylate. The two reactions share an active site; however, the former reaction is approximately 2 orders of magnitude less efficient than the latter.^11^ In the first reported QM/MM study on the entropic contributions of PchB, Xie et al. used DFTB2/MIO/MM to calculate thermodynamic activation parameters for the conversion of chorismate to pyruvate in PchB, finding a significantly less negative activation entropy than that observed in the uncatalyzed reaction.^12^ By including a correctional term obtained from quasiharmonic analysis, they could match experimental entropy values. However, the activation free energies of the enzymatic and aqueous reactions reported by Xie et al. are also significantly lower than the experimental values, indicating that their calculated entropy from the Arrhenius plot approach is likely incorrect.

In comparison to other pyruvate lyases in the shikimate pathway, PchB is more similar to CMs of the AroQ class such as EcCM from *Escherichia coli*, both structurally and functionally.^10,13,14^ The apparent origin of PchB is a gene duplication of an AroQ CM requiring a few minor mutations in the active site for efficient isopyruvate lyase activity, influencing a reduction in CM activity.^11^ Mutation of the positively charged Arg90 to the non-natural neutral amino acid citrulline in BsCM decreased the catalytic rate significantly, with only a modest decrease in *K*_M_, suggesting that electrostatic stabilization plays a key role in the CM catalytic activity.^15,16^ In EcCM, Val35Ile and Val35Ala mutations result in an increase in *k*_cat_ of about 1.5 times and decrease in *k*_cat_ by about 2 times, respectively, reflecting an Ala35Ile mutation, which in EcCM introduces an overall increase in *k*_cat_ by about 3 times.^17^ The lower enzymatic efficiency of PchB compared with that of BsCM has been suggested to be due to a less restricted effect on the substrate in the former. Thus, an equivalent Ala38Ile mutation in the active site of PchB contributes to a significant increase in *k*_cat_, as it results in a better electrostatic stabilization for the transition state.^5^ This suggests that enzyme efficiency is increased by favoring active sites which contribute to substrate preorganization, thereby reducing the entropic penalty paid. In both AroQ- and AroH-class CMs, preorganization of the substrate into a compressed conformer appears to be a key component in making the activation entropy less negative and therefore decreasing the activation free energy in the enzyme-catalyzed reaction. Thus, a further understanding of the phase space of the substrate conformation is crucial in elucidating the role of enthalpy and entropy in the reaction.

Understanding the roles that enthalpy and entropy play in enzymatic reactions requires an intimate knowledge of the microscopic details underlying these reactions. Due to difficulties in obtaining atomistic levels of detail in enzymatic reactions, making use of computer simulations provides an optimal route to study the details of the activation enthalpy–entropy balance of such systems. In this study, we employ semiempirical EVB/MD free energy calculations to study the CM reaction in PchB and BsCM. EVB was chosen due to its ability to accurately calculate free energies while being less computationally intensive than ab initio QM/MM methods.^18−20^ Furthermore, EVB can be combined with an Arrhenius plot to extract the thermodynamic activation enthalpies and entropies. In our work, simulations are performed at six evenly spaced temperatures ranging from 288 to 313 K, allowing for calculation of the thermodynamic activation parameters from an Arrhenius plot.^2^

## 2 Methods

### 2.1 DFT Calculations

The reaction energies for the uncatalyzed transformation of chorismate to prephenate in water were calculated with DFT using Gaussian 16.^21^ Structure optimization and frequency calculations at the reactant (chorismate), transition, and product (prephenate) states were performed with the B3LYP functional^22^ and the 6-31G(d,p) basis set. Dispersion effects were included in all calculations using Grimme’s B3LYP-D3 method.^23,24^ Intrinsic reaction coordinate calculations were performed in both directions from the TS to verify that the correct minima are connected. Electronic energies were obtained from single-point calculations on the optimized geometries (RS, TS, and PS) at the B3LYP/6-311G+(2d,2p) level of theory. The final energies reported are corrected for zero-point energy (ZPE), dispersion, and solvation effects calculated with the CPCM^25^ model using water as the solvent (eps = 80). The same procedure was also performed with the M06-2X^26^ functional for comparison. In contrast to B3LYP, dispersion corrections are already included in the M06-2X functional; therefore, this was not added explicitly. Additional calculations were also performed with both functionals using the SMD^27^ solvation model parameterized for water.

### 2.2 EVB Simulations

The crystal structures of PchB (PDB entry 3REM)^28^ and BsCM (PDB entry 3ZO8)^15^ were used as starting models for the EVB simulations. For PchB, the chorismate was manually built in to the active site by modifying the existing salicylate and pyruvate present in the active site of 3REM.^28^ The chorismate configuration for BsCM was obtained by manually modifying the transition state inhibitor present in the Arg90Cit BsCM with PDB entry 3ZP4(15) and then superimposed to the wild-type BsCM (PDB entry 3ZO8).^15^ Topologies for all simulations were prepared with Q^29^ and Qgui^30^ where parameters were assigned in accordance with the OPLS-AA/M force field.^31^ The enzyme structures were solvated in a spherical droplet of TIP3P^32^ water molecules with a simulation sphere radius of 36 Å, centered at the protein center of mass and encompassing the entire protein. A 25 Å radius was used for the chorismate to prephenate reference reaction in water. The systems were equilibrated prior to the MD/EVB simulations by gradually increasing the temperature from 1 to the final temperature (283–308 K) through a stepwise scheme (six steps). The first five steps were simulated for 10 ps, whereas the final equilibration step was simulated for 100 ps with a time step of 1 fs. The temperature was controlled by coupling the system to an external bath,^33^ with a relaxation time of 10 fs for the first five steps and 100 fs for the final equilibration step and the productive phase. Long-range electrostatics were treated using the multipole expansion method (LRF)^34^ and a direct 10 Å cutoff for nonbonded interactions. The reacting fragments were, however, allowed to interact with the entire system (no cutoff). Bonds and angles of solvent molecules were constrained with the SHAKE algorithm.^35^ The productive simulations comprised 51 discrete free energy perturbation (FEP) steps, where each step was simulated for 100 ps, resulting in 5100 ps for each run.

The EVB free energy profiles were calculated using the FEP umbrella sampling approach described elsewhere.^37,38^ These calculations utilized 51 discrete FEP windows, with a constant spacing of 0.02, between the two end-points chorismate (λ = 0) and prephenate (λ = 1). The EVB Hamiltonian was parameterized by fitting the relevant parameters (Δα and H_12_) for the uncatalyzed transformation of chorismate to prephenate in water to reproduce the energetics obtained with DFT. That is, the activation free energy, Δ*G*^⧧^, was fitted to 24.5 kcal/mol, which is also the experimentally determined barrier,^36^ whereas the reaction free energy, Δ*G*_0_, was fitted to −12.8 kcal/mol, which is in excellent agreement with the previously estimated Δ*G*_0_ from the observed reaction enthalpy, Δ*H*_0_ = −13.3 kcal/mol, utilized by Warshel et al.^3^ Thermodynamic activation parameters were obtained from EVB/Arrhenius plots, as explained elsewhere^37,38^ based on simulations at six different temperatures evenly spaced from 288 to 313 K. A total of 100 independent simulations were carried out at each temperature point, resulting in 600 individual EVB simulations (306 ns) each for PchB, BsCM, and the uncatalyzed reaction (1800 simulations/918 ns in total).

## 3 Results and Discussion

### 3.1 EVB and DFT Modeling of the Reference Reaction

**Figure 2:**
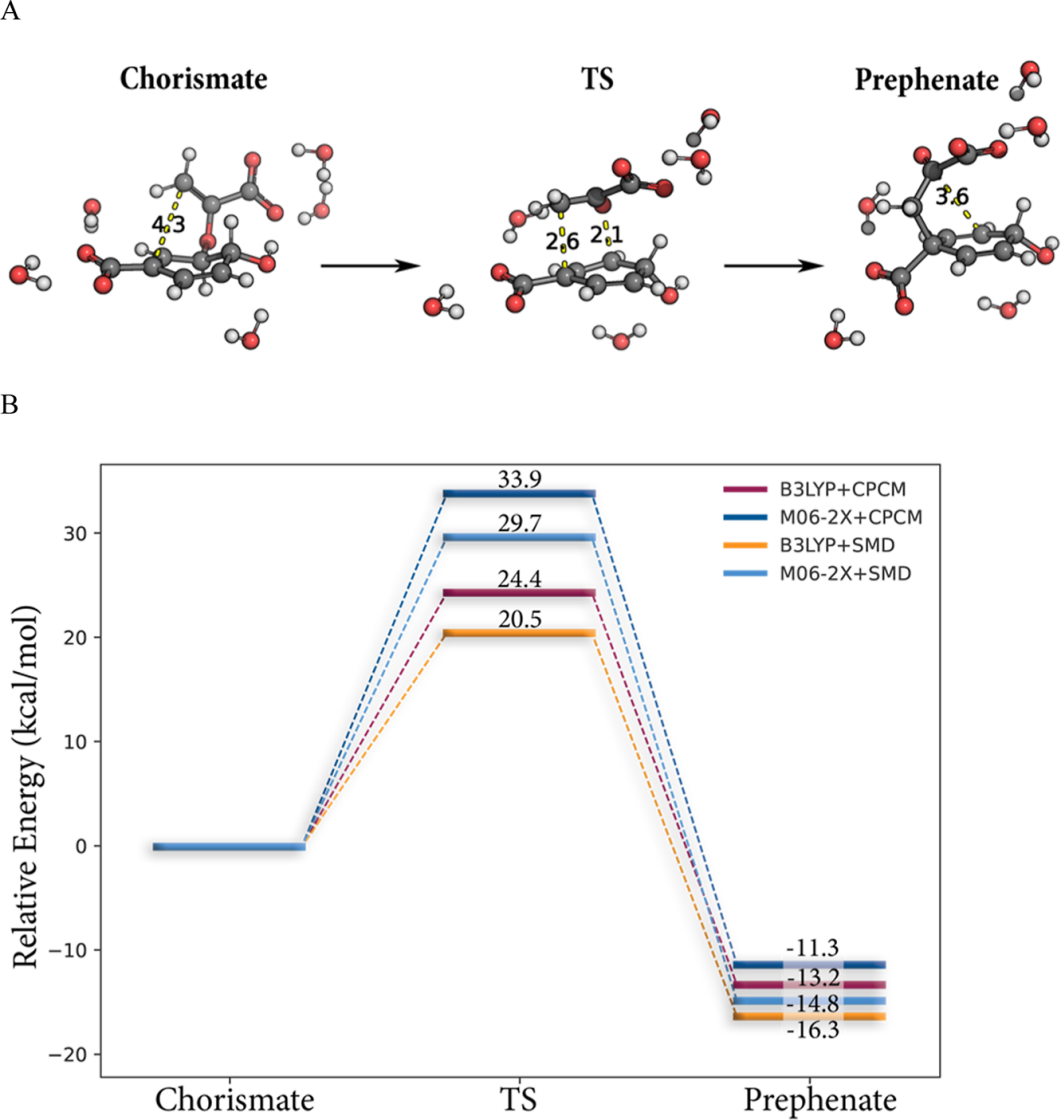
(A) DFT/B3LYP-optimized geometries for the conversion of the chorismate reactant state to the prephenate product state and (B) comparison of the calculated energetics with the B3LYP and M06-2X functionals including zero-point energy and solvation corrections obtained from the CPCM and SMD solvation models.

Calibrating the EVB Hamiltonian requires that both activation and reaction free energies are available, from either experiments or quantum mechanical calculations. The activation free energy for the uncatalyzed transformation of chorismate to prephenate has previously been experimentally determined to be 24.5 kcal/mol,^36^ but the reaction free energy, Δ*G*_0_, is not available. Thus, to obtain a reference for Δ*G*_0_, we carried out DFT calculations for the unimolecular conversion of chorismate to prephenate in water (Figure 2). To stabilize the charged carboxylic acid groups on the chorismate, five explicit water molecules were included. The B3LYP/6-311G+(2d,2p) model^39^ with CPCM solvation^40,41^ correction yields a barrier of 24.4 kcal/mol, which is in excellent agreement with the corresponding experimental result.^36^ Moreover, the reaction free energy was calculated to be −13.2 kcal/mol, which is very close to the experimentally observed reaction enthalpy, Δ*H*_0_ = −13.3 kcal/mol, which was also previously used by Warshel et al. to estimate Δ*G*_0_ for this reaction.^3^

Even though the CPCM model is often considered one of the most successful solvation models,^25,42^ the more recent SMD model developed by Marenich et al.^27^ is also popular, perhaps more commonly in combination with the Minnesota functionals developed by the same group. Thus, for comparison, we ran the same calculations with B3LYP and SMD, as well as M06-2X with CPCM and SMD (Figure 2B). As illustrated in Figure 2B, the resulting spread of the predicted activation energy is large. The difference between the B3LYP and M06-2X barriers is as much as 9.5 and 9.2 kcal/mol with the CPCM and SMD solvation correction, respectively. This difference is surprisingly large for the small unimolecular reaction under investigation here, demonstrating the importance of awareness of functional dependencies. However, the main aim of our DFT calculations was to obtain the reaction free energy to be used for calibrating the EVB Hamiltonian, and here, the variation is within the expected error of DFT calculations (Figure 2B). The difference between B3LYP and M06-2X reaction energies here is only 1.5 and 1.9 kcal/mol with the CPCM and SMD solvation corrections, respectively. The fact that the B3LYP calculations with CPCM reproduce both the experimentally known barrier and a reaction energy in agreement with the experimental reaction enthalpy suggests that −13.2 kcal/mol is a sufficiently reliable estimate for Δ*G*_0_. Thus, this value was used further to calibrate the EVB reference reaction Hamiltonian. One should also note that the explicit water molecules used ensure a more proper microscopic solvation of the charges, and the orientation and the overall geometry are virtually identical when comparing the transition state obtained with B3LYP and M06-2X (Figure S1). The barrier calculated without these explicit water molecules is consistently 0.6–1.7 kcal/mol lower, depending on the functional and solvation correction, but the transition-state geometry is virtually identical compared to the calculations with explicit water (Figure S1).

While DFT methods offer a good combination of accuracy and computational cost, it should be noted that the variation observed in the activation free energy with different functionals and solvation models is a well-known problem with DFT calculations. It thus becomes difficult to trust which result should be used. A remedy to alleviate the functional dependency observed in the calculated activation free energies has been suggested, which is to use correlated ab initio electronic structure methods such as coupled cluster in multiscale modeling methods.^43^ Here, Mulholland and co-workers showed that the more than 13 kcal/mol difference in the activation free energy was removed by using projector-based embedding.^43^ However, for this study, the experimental barrier is known, and the thermodynamic activation parameters are not very sensitive to small changes in the reaction free energy.

EVB calculations of the thermodynamic activation parameters for the conversion of chorismate to prephenate were first performed on the uncatalyzed reference reaction in an aqueous environment. Reactions at 298 K were fit to the experimental value of the activation free energy (24.5 kcal/mol) and the reaction free energy obtained from DFT (−13.2 kcal/mol), yielding the intrinsic gas-phase energy difference between the EVB resonance structures, Δα = −94.7, and the off-diagonal coupling element, *H*_12_ = 86.8 kcal/mol. Using these parameters, EVB/MD calculations were performed at six other temperatures to obtain an Arrhenius plot of the solution-phase reaction. From this plot, an activation free energy of Δ*G*^⧧^ = 24.4 kcal mol^–1^ was obtained, and a linear regression was performed, yielding activation enthalpies and entropies of Δ*H*^⧧^ = 20.9 kcal mol^–1^ and *T*Δ*S*^⧧^ = −3.4 kcal mol^–1^ (at 298 K), respectively. These parameters are in excellent agreement with the values obtained from the experiment (Table 1).

**Table 1:** Thermodynamic Activation Parameters (in kcal/mol at 298 K) for the CM Reaction (Chorismate → Prephenate) in Water (Uncatalyzed) and Catalyzed by Isochorismate Pyruvate Lyase from *Pseudomonas aeruginosa* (PchB) and in CM from the *B. subtilis* (BsCM), Respectively^a^

	Δ*G*^⧧^	Δ*H*^⧧^	*T*Δ*S*^⧧^
Uncatalyzed			
experimental^b^	24.5	20.7	–3.8
DFT (this study)	24.4		
DFTB2/MIO^c^	15.6 ± 0.2		–2.6
EVB (this study)	24.4 ± 0.6	20.9 ± 0.4	–3.4 ± 0.4
PchB			
experimental^d^	19.5	15.9 ± 0.2	–3.6 ± 0.2
DFTB2/MIO^c^	12.1 ± 0.1		–1.0
EVB (this study)	18.3 ± 0.7	14.0 ± 0.5	–4.3 ± 0.5
BsCM			
experimental^e^	15.4	12.7 ± 0.4	–2.7 ± 0.4
EVB (this study)	15.8 ± 12.1	12.9 ± 0.3	–2.9 ± 0.3

a Reported errors are given as standard error of the mean.

b Andrews et al.^36^

c Xie et al.^12^

d Lamb et al.^10^

e Kast et al.^46^

### 3.2 Activation Parameters of CM Enzyme Reactions

**Figure 3:**
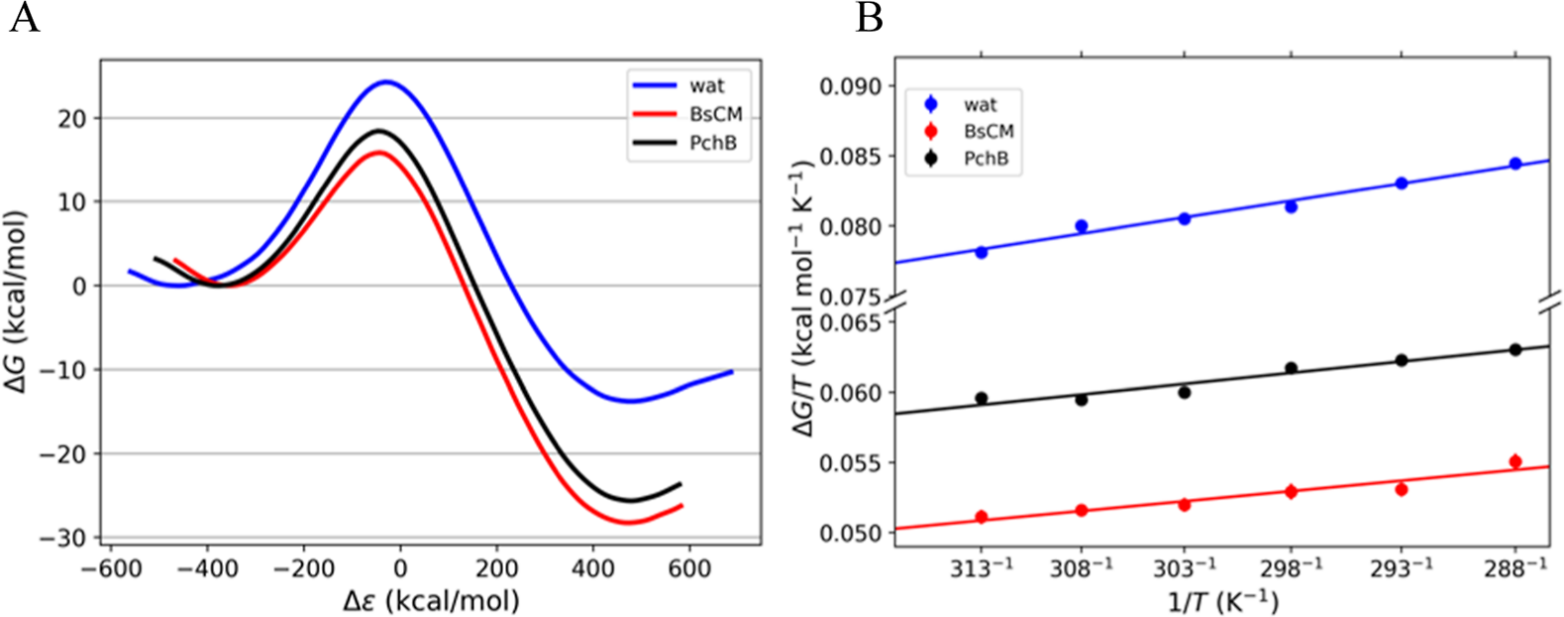
(A) Reaction free energy profiles obtained from EVB simulations at 298 K and (B) the corresponding Arrhenius plots in the temperature range of 288–313 K for the conversion of chorismate to prephenate in an aqueous environment (blue) and in the CM enzymes from *B. subtilis* (red) and PchB (black). Error bars are displayed but are smaller than the data point.

Before constructing the Arrhenius plots for the CM enzymes, we first looked at the thermodynamic behavior at 298 K. The calculated average reaction free energy profile along the reaction coordinate for each enzyme (Figure 3A) yielded free energy barriers of Δ*G*^⧧^ = 15.8 kcal mol^–1^ for BsCM and Δ*G*^⧧^ = 18.3 kcal mol^–1^ for PchB, which show substantial catalysis compared to the uncatalyzed reference reaction activation barrier of Δ*G*^⧧^ = 24.4 kcal mol^–1^. Activation free energies for both enzymatic reactions are also in good agreement with the experimental values for the respective enzymes.^10,46^

To obtain thermodynamic activation parameters from an Arrhenius plot (Figure 3B), EVB/MD calculations were performed at the same six temperatures used for the reference reaction, using the same Δα and *H*_12_ values obtained from fitting the reference reaction to activation and reaction free energies of the uncatalyzed reaction obtained from experiment^36^ and DFT. From these simulations, we fitted Δ*G*^⧧^/*T* vs 1/*T* using linear regression, which yielded values of Δ*H*^⧧^ = 12.9 kcal mol^–1^ and *T*Δ*S*^⧧^ = −2.9 kcal mol^–1^ for BsCM at 298 K. The same analysis for PchB yielded values of Δ*H*^⧧^ = 14.0 kcal mol^–1^ and *T*Δ*S*^⧧^ = −4.3 kcal mol^–1^ at 298 K. The values predicted using the Arrhenius plot approach for both enzymes are thus in a very reasonable agreement with the experimental values of Δ*H*^⧧^ = 12.7 kcal mol^–1^ and *T*Δ*S*^⧧^ = −2.7 kcal mol^–1^ for BsCM at 298 K, and Δ*H*^⧧^ = 15.9 kcal mol^–1^ and *T*Δ*S*^⧧^ = −3.6 kcal mol^–1^ for PchB at 298 K.

For the CM reaction catalyzed by PchB, an earlier computational study has been published by Xie et al.^12^ They report activation parameters of Δ*G*^⧧^ = 12.1 kcal mol^–1^ and *T*Δ*S*^⧧^ = −1.0 kcal mol^–1^ and claimed that since their calculated enzyme activation entropy penalty is 1.6 kcal/mol smaller than that of the uncatalyzed reaction, the enzyme-catalyzed reaction is entropically driven. However, their reported activation free energy is also significantly lower than experimental values.^10^ In comparison to the enzyme-catalyzed reaction, we obtain activation parameters of Δ*H*^⧧^ = 20.9 and *T*Δ*S*^⧧^ = −3.4 kcal mol^–1^ K^–1^ for the reference reaction in water, in excellent agreement with experiment.^36^ The enzyme-catalyzed reactions in BsCM and PchB thus show a significant reduction in the activation enthalpy, while reductions in the entropy penalty are much smaller. Hence, both from our calculations and experiment, it can be concluded that the enzyme-catalyzed reactions are enthalpy driven and not entropy driven as suggested by Xie et al.^12^

A better understanding of the difference in activation thermodynamics can be obtained from a breakdown of the energetics by analyzing the MD trajectories. Neglecting the small pressure–volume term, the activation enthalpy can be approximated by
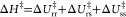
1where the subscripts r and s denote the reacting molecule (chorismate/prephenate) and its surrounding environment, respectively. Here, the last Δ*U*_ss_^⧧^ term involves numerically very large energies since it corresponds to the interactions within the entire protein/solvent system. However, the average Δ*U*_rr_^⧧^ and Δ*U*_rs_^⧧^ terms can be readily evaluated since they only involve interactions with and within the reacting molecule, and the difference is then taken between the transition and reactant states. It is also of interest here to further break down the contribution from the reacting molecule into its bonded (bonds, angles, torsions, and improper dihedrals) and nonbonded (electrostatic and van der Waals) terms
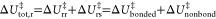
2

**Figure 4:**
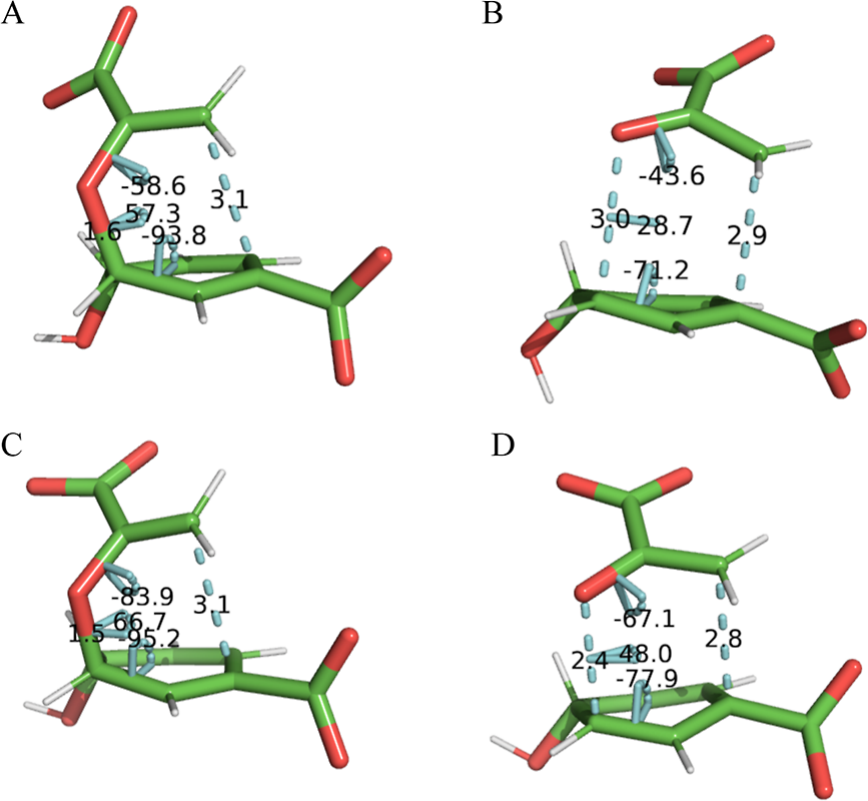
Geometries of chorismate displaying C_5_–O_7_ and C_1_–C_9_ distances and C_1_–C_6_–C_5_–O_7_, C6–C_5_–O_7_–C_8_, and C_5_–O_7_–C_8_–C_9_ torsions for the uncatalyzed RS (A) and TS (B), as well as the BsCM RS (C) and TS (D).

Such a breakdown (Table 2) clearly shows that the trend in Δ*H*^⧧^ obtained from the Arrhenius plots follows the contribution from Δ*U*_tot,r_^⧧^. Hence, the value of Δ*U*_tot,r_^⧧^ compared to the uncatalyzed reaction is about 10 kcal/mol lower for PchB and about 20 kcal/mol lower for BsCM. The magnitude of Δ*U*_tot,r_^⧧^ is mainly determined by the several partially formed (single and double) bonds in the transition state, which is the reason why its main contribution comes from Δ*U*_bonded_^⧧^. Here, one also finds that the two main contributions to the differences in Δ*U*_bonded_^⧧^ compared to the water reaction (Table 2) originate from the Morse potential bond terms (∼10 kcal/mol) and the torsional energies (∼4 kcal/mol), while angle and improper terms have negligible contributions. Hence, both enzymes enforce a more compact transition state than in water, which results in more favorable values of Δ*U*_bonded_^⧧^. As seen in Figure 4, the C_5_–O_7_ and C_1_–C_9_ distances are compressed in both the BsCM RS and the TS in comparison to the uncatalyzed reaction. Furthermore, the C_1_ and C_9_ atoms are better aligned in the BsCM RS due to differences in the C_1_–C_6_–C_5_–O_7_, C_6_–C_5_–O_7_–C_8_, and C_5_–O_7_–C_8_–C_9_ torsions in comparison to the uncatalyzed RS. The changes in these bond lengths and torsions are largely responsible for the more favorable Δ*U*_bonded_^⧧^ seen in BsCM. Interestingly, one finds that the average value of Δ*U*_nonbond_^⧧^ is actually less favorable in the promiscuous PchB enzyme than in both water and BsCM. This is likely due to poorer substrate preorganization as the CM reaction is not the primary function of this enzyme.

**Table 2:** Decomposition of Total Potential Activation Energy into Bonded and Nonbonded Contributions (in kcal/mol at 298 K) for the CM Reaction (Chorismate → Prephenate) in Water (Uncatalyzed) and Catalyzed by Isochorismate Pyruvate Lyase from *Pseudomonas aeruginosa* (PchB) and in CM from the *B. subtilis* (BsCM), Respectively

	Δ*U*_tot,r_^⧧^	Δ*U*_bonded_^⧧^	Δ*U*_nonbond_^⧧^
uncatalyzed	111.0	110.0	1.0
PchB	101.8	96.0	5.8
BsCM	89.3	93.2	–3.9

### 3.3 Structural Analysis of the Catalytic Effect of CMs

**Figure 5:**
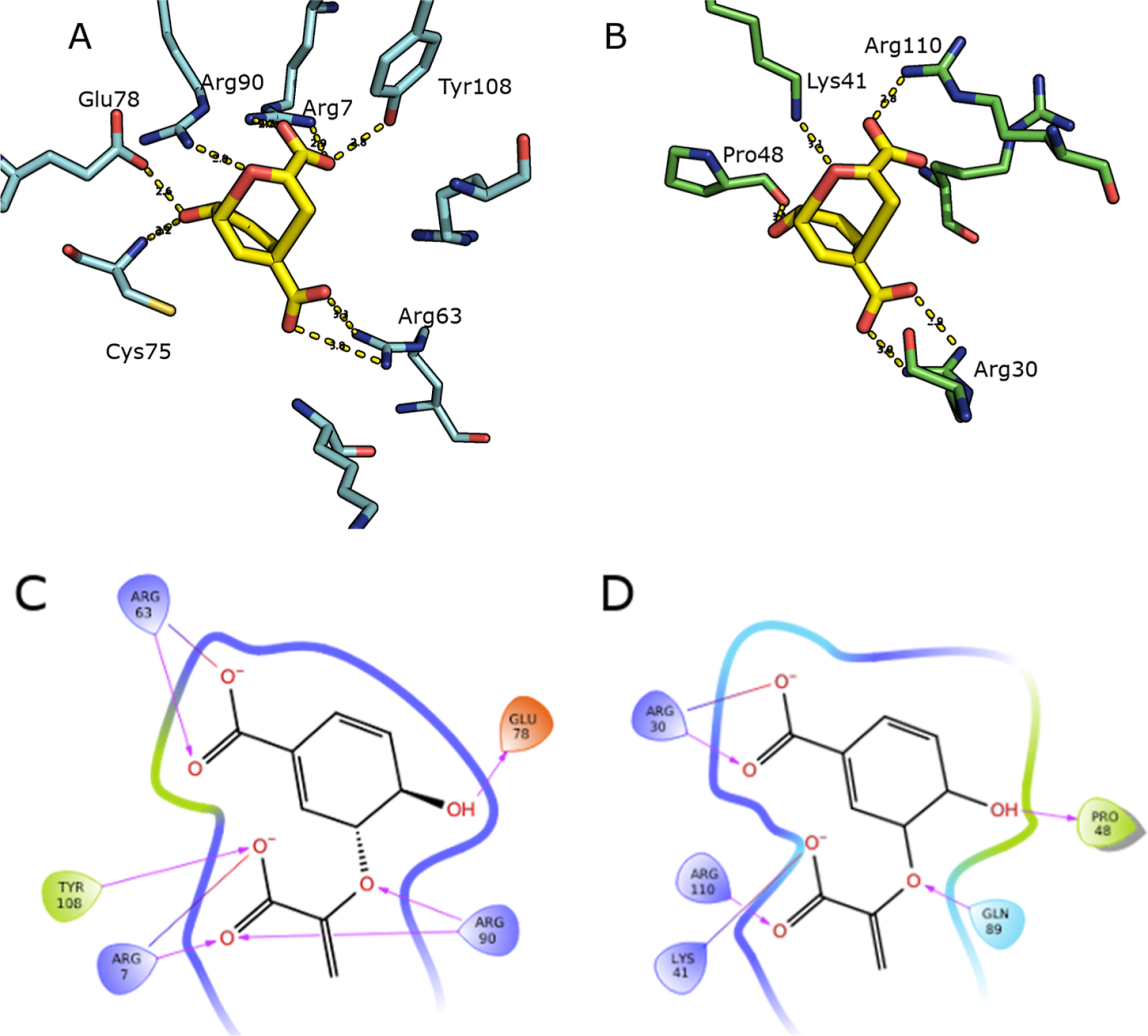
Active sites of CM enzymes from simulated MD trajectory snapshots of the CM reaction in BsCM and PchB showing residues of interest in catalysis. (A) Three-dimensional active site with a simulated pericyclic transition state in *B. subtilis.* (B) Three-dimensional active site with a simulated pericyclic transition state in PchB. (C) Two-dimensional ligand interaction diagram of BsCM. (D) Two-dimensional ligand interaction diagram of PchB.

The enthalpically driven nature of catalysis by the CM enzymes BsCM and PchB can be attributed to highly charged active sites, which serve to stabilize the transition state, as seen in Figure 5. It is notable that in comparison to PchB, the active site of BsCM has a greater degree of hydrogen and ionic bonding between the substrate and active site residues, which contribute to the lower enthalpy seen in BsCM. In particular, there are three key residues in each enzyme that contribute to this effect: Glu78, Arg90, and Tyr108 in BsCM, and Pro48, Lys41, and Arg110 in PchB. Additionally, the second carboxylate of the substrate is stabilized by Arg63 in BsCM and Arg30 in PchB.

Strong hydrogen bonding between Glu78 and C_4_–OH has been proposed to cause an elongation of the C_5_–O_7_ bond, stabilizing the transition state in *B. subtilis*.^44^ In *P. aeruginosa*, the backbone carbonyl group of Pro48 serves a similar function, as it acts as a hydrogen bond acceptor in forming a hydrogen bond with C_4_–OH. However, the hydrogen bond formed in PchB is at a somewhat unfavorable angle for this interaction, thus suggesting why the enthalpic effect is greater in BsCM where the anionic oxygen is also stabilized by the amide group of Cys75. This is supported by mutagenesis studies showing that mutations of this glutamate to residues reducing this interaction result in a reduction of the catalytic rate.^45^ Furthermore, the elongation of this C_5_–O_7_ ether bond by Glu78/Cys75 (in BsCM) or Pro48 (in PchB) results in a large negative charge on the ether oxygen. In BsCM, Arg90 provides a positive charge to counterbalance the large negative charge on this ether oxygen. Lys41 in PchB also provides a positive charge to stabilize the negative charge on this oxygen. To ensure the proper formation of the prephenate product, Arg7 and Tyr108 in BsCM form hydrogen bonds with the carboxylate group in the pyruvate moiety of the transition state. Similarly, Arg110 and Lys41 serve this function in PchB. The ionic interactions are essential in the CM reaction, as they orient the pyruvate fragment with the cyclohexadienyl moiety such that it facilitates the formation of the C_1_–C_9_ bond in prephenate. The differences in the active sites between the two enzymes can thus explain the decrease in the activation enthalpy seen in BsCM in comparison to that seen in PchB. Enhanced hydrogen bonding in the active site of BsCM results in more favorable internal energetics within the reacting atoms and between the reacting atoms and their surroundings.

## 4 Concluding Remarks

EVB/MD simulations were used here to determine thermodynamic activation parameters for the conversion of chorismate to prephenate, in both the uncatalyzed reaction and the CM enzymes BsCM and PchB. The success of the EVB method is demonstrated by its ability to accurately match thermodynamic activation parameters obtained from experiments in all cases. These results clearly support the notion that the CM enzymes BsCM and PchB are enthalpy-driven reactions, as seen by the substantial reduction in activation enthalpies and smaller reductions in activation entropy in comparison to that observed in the uncatalyzed reaction.

Understanding the roles that entropy and enthalpy play in enzyme catalysis is crucial in understanding enzyme function as a whole. In particular, we are interested in the temperature dependence of chemical reaction rates and how enzymes have evolved to function efficiently at the freezing point of water. The present study provides knowledge of the activation parameters for BsCM and PchB that will serve as a starting point for studying the shift in the entropy–enthalpy balance for cold-adapted CM enzymes.

## Supporting Information Available

Optimized transition state geometries and their coordinates (PDF)
