# Supplementary material for: Accurate Computation of Thermodynamic Activation Parameters in the Chorismate Mutase Reaction from Empirical Valence Bond Simulations
Source: J Chem Theory Comput. 2023 Dec 19;20(1):451–8. doi: 10.1021/acs.jctc.3c01105 (PMC10782440; doi:10.1021/acs.jctc.3c01105)
Supplement: Supplementary file 1 — ct3c01105_si_001.pdf [file ct3c01105_si_001.pdf]

Supporting Information

Accurate computation of thermodynamic activation parameters in the chorismate mutase reaction from empirical valence bond simulations

*Ryan Scott Wilkins, Bjarte Aarmo Lund, Geir Villy Isaksen, Johan Åqvist and Bjørn Olav Brandsdal**

Hylleraas Centre for Quantum Molecular Sciences, Department of Chemistry, University of Tromsø, N9037 Tromsø, Norway

Corresponding Author

* Bjørn Olav Brandsdal - Hylleraas Centre for Quantum Molecular Sciences, Department of Chemistry, University of Tromsø, N9037 Tromsø, Norway; https://orcid.org/0000-0002-4681-8081; Email: bjorn-olav.brandsdal@uit.no

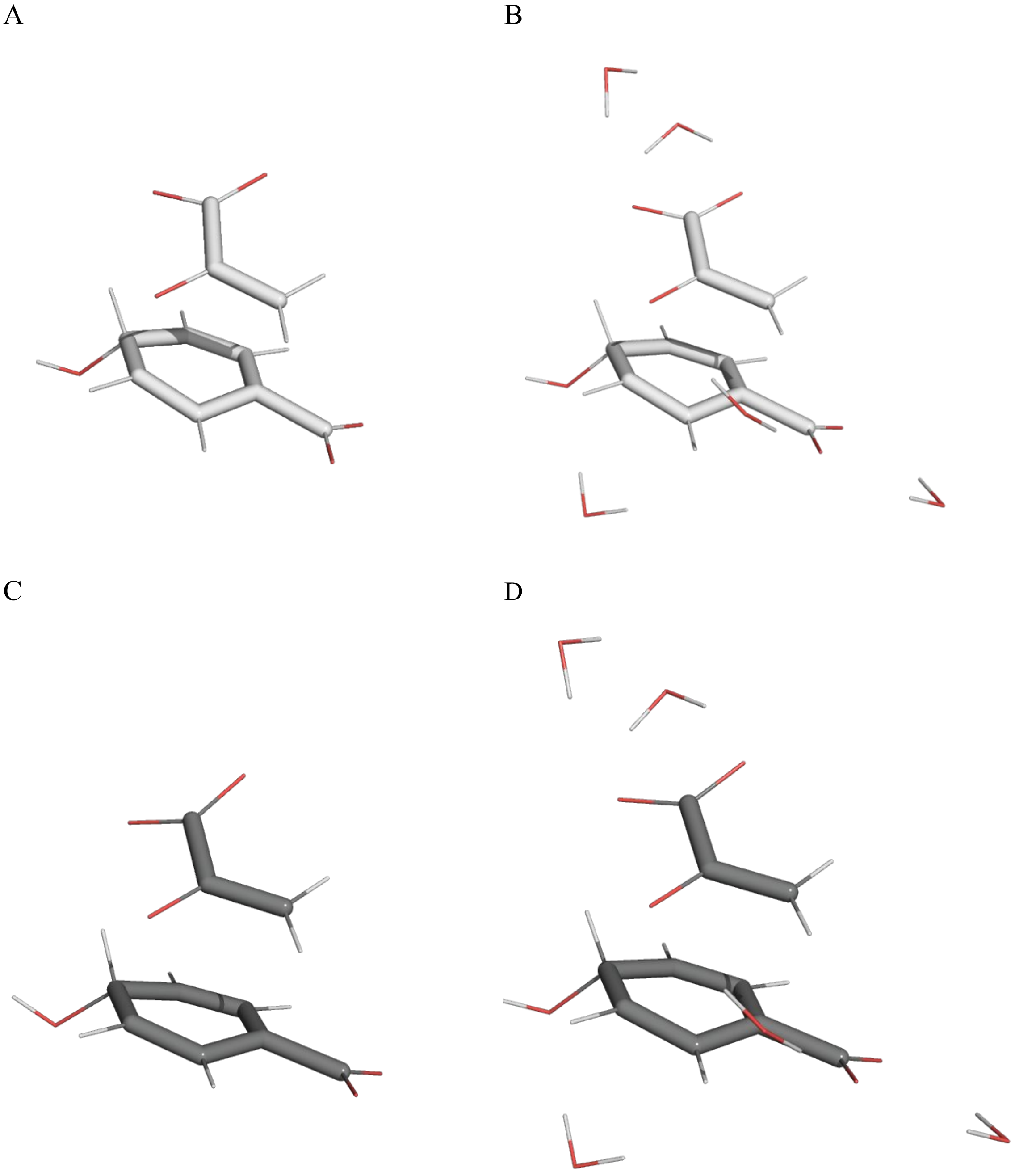

A
B
C
D

Figure S1: Optimized transition state geometries of the chorismate mutase reaction from DFT using M06-2X (A – without explicit water, B – with explicit water) and B3LYP (C – without explicit water, D – with explicit water) functionals.

Optimized transition state Cartesian coordinates (in Å) of the chorismate mutase reaction from DFT using M06-2X (A – without explicit water, B – with explicit water) and B3LYP (C – without explicit water, D – with explicit water) functionals.

M06-2X (no explicit water)

C	1.33690900	0.30918900	0.11468000
C	0.55611900	0.40860800	1.36151500
C	0.93983600	1.10987400	-0.95140300
C	-0.61224500	1.05515400	1.41473200
H	1.00714600	-0.06182300	2.22937600
C	2.81699600	-0.16765000	0.19577000
C	-0.40985200	1.43438500	-1.05148500
H	1.60007300	1.15246900	-1.81222400
C	-1.26830600	1.61512700	0.19056200
H	-1.17791200	1.13781200	2.33964400
O	3.23842200	-0.41098400	1.34823500
O	3.41698600	-0.23451300	-0.90125700

H	-0.75777800	2.00999200	-1.90992300
O	-1.54463600	3.02170300	0.41150600
H	-2.20724800	1.06542700	0.03877200
H	-2.24072100	3.23869500	-0.21757100
C	0.47964700	-1.62642700	-0.74275400
C	-0.82854100	-1.16748800	-0.83969200
H	0.68756200	-2.39959400	-0.01114700
H	1.21322300	-1.44607600	-1.51893000
C	-1.90766400	-1.66877700	0.17564700
O	-1.12743900	-0.22442300	-1.67313900
O	-2.94285000	-0.96646500	0.27766900
O	-1.63320100	-2.74892500	0.74405700

M06-2X (explicit water)

C	-1.38338200	0.01120900	-0.41132400
C	-0.48335700	0.20059500	-1.56608500
C	-1.38686200	1.00418000	0.56524100
C	0.48114900	1.12521100	-1.55904800
H	-0.66142600	-0.44957900	-2.41612400
C	-2.63864600	-0.86363400	-0.57117500
C	-0.18911100	1.68281200	0.79234500
H	-2.16059700	0.99512900	1.32404400

C	0.77271000	1.96328300	-0.35129200
H	1.13435900	1.27267900	-2.41410700
O	-2.86385600	-1.30397600	-1.71569800
O	-3.31917500	-1.03657100	0.47931000
H	-0.14609600	2.41428300	1.59756900
O	0.68673100	3.35568900	-0.74491600
H	1.79066000	1.74685300	-0.00727500
H	1.12332300	3.86255700	-0.05042800
C	-0.18974300	-1.55609600	0.69808500
C	0.88465900	-0.71590200	0.94826500
H	-0.03923400	-2.37547300	0.00381300
H	-1.02723500	-1.61763500	1.37844600
C	2.19588600	-0.90352100	0.14738200
O	0.75247400	0.31499100	1.71755600
O	3.04819700	0.02634800	0.22307000
O	2.30131200	-1.98188300	-0.47715200
O	-2.31561100	-0.58415700	3.12785400
H	-1.42302600	-0.29515300	2.89486900
H	-2.72670700	-0.76446100	2.25749000
O	5.61745800	-0.96782700	1.05443100
H	4.74348700	-0.54415500	1.09139200

H	5.56852600	-1.34518300	0.16134200
O	4.69907900	-1.33101300	-1.63760400
H	3.90937400	-1.84800600	-1.36430000
H	4.40818400	-0.48836800	-1.24900900
O	-2.01737700	4.11617100	-0.22042300
H	-1.13837100	3.92589900	-0.58704100
H	-2.35830100	3.22619800	-0.06862900
O	-5.32087900	-2.49012000	-0.93377800
H	-4.60278600	-2.25285800	-1.54534800
H	-4.92077900	-2.09275500	-0.14225600

B3LYP (no explicit water)

C	1.49069500	0.30056900	0.11578500
C	0.79499400	0.62399400	1.35769000
C	1.04797000	0.91023000	-1.05930300
C	-0.33514500	1.35299200	1.37163900
H	1.27114600	0.26397600	2.26424800
C	2.90114300	-0.34267600	0.17185000
C	-0.23016000	1.43369200	-1.12953700
H	1.65130600	0.76334100	-1.94946800
C	-1.04337700	1.73405200	0.11066100

H	-0.84410900	1.60473500	2.29971500
O	3.39229000	-0.46533800	1.32599100
O	3.40786400	-0.64460000	-0.94111500
H	-0.58694200	1.90524600	-2.04129200
O	-1.41280300	3.15587700	0.15508700
H	-1.96569300	1.12259600	0.07132300
H	-2.27311800	3.18791600	-0.28146400
C	0.19320400	-1.89993100	-0.55756200
C	-1.02701000	-1.25626200	-0.73903800
H	0.29412900	-2.60290700	0.26061400
H	1.00607200	-1.80689400	-1.26652600
C	-2.18688900	-1.50661500	0.29211500
O	-1.20513200	-0.40158100	-1.68774900
O	-3.03735900	-0.58018400	0.42196100
O	-2.16802800	-2.63145900	0.85545700

B3LYP (explicit water)

C	-1.51810800	0.13177900	-0.45241000
C	-0.64155100	0.36579300	-1.60056300
C	-1.40107800	0.97822200	0.64839400
C	0.37535900	1.24215800	-1.54842800
H	-0.87420600	-0.19933700	-2.49652700

C	-2.76788400	-0.74224500	-0.60023500
C	-0.20658500	1.65604700	0.85621000
H	-2.12570300	0.90515300	1.44850500
C	0.74842400	1.94711600	-0.28197600
H	1.01621200	1.42457300	-2.40604500
O	-3.08031700	-1.06903400	-1.77228600
O	-3.37485400	-1.03517800	0.47651900
H	-0.08965000	2.28571400	1.73309100
O	0.78119600	3.39153100	-0.55739900
H	1.75516800	1.62136100	0.01626800
H	1.29390900	3.78689100	0.16095400
C	-0.11003400	-1.77670100	0.58930200
C	0.91849500	-0.90421600	0.90541600
H	0.06183000	-2.53273200	-0.16643800
H	-1.00321900	-1.84200200	1.19116900
C	2.25657300	-1.00224500	0.12037900
O	0.76362600	0.04744900	1.76944700
O	3.03841500	-0.00337400	0.18023400
O	2.45926600	-2.08555700	-0.48937600
O	-2.20802300	-0.84419100	3.10311300
H	-1.30727300	-0.58235500	2.85769600
H	-2.65493000	-0.94114700	2.23394500

O	5.72112300	-0.76655800	0.93724800
H	4.80997000	-0.41975000	0.97738700
H	5.67634000	-1.18590000	0.05844800
O	4.77457700	-1.33201500	-1.72975200
H	4.00646700	-1.85110900	-1.37975300
H	4.47104000	-0.46970900	-1.39616500
O	-1.89240000	4.25251600	-0.04612200
H	-0.98825600	4.06202200	-0.35959200
H	-2.24923600	3.35874900	0.04963900
O	-5.47102500	-2.38806800	-0.94958900
H	-4.79048200	-2.06436100	-1.57126800
H	-5.02231800	-2.07046600	-0.14414900
